# A muscle-driven approach to restore stepping with an exoskeleton for individuals with paraplegia

**DOI:** 10.1186/s12984-017-0258-6

**Published:** 2017-05-30

**Authors:** Sarah R. Chang, Mark J. Nandor, Lu Li, Rudi Kobetic, Kevin M. Foglyano, John R. Schnellenberger, Musa L. Audu, Gilles Pinault, Roger D. Quinn, Ronald J. Triolo

**Affiliations:** 1 Department of Veterans Affairs, Advanced Platform Technology Center, Louis Stokes Cleveland VA Medical Center, 10701 East Blvd, 151AW/APT, Cleveland, OH 44106 USA; 2 Department of Biomedical Engineering, Case Western Reserve University, 10900 Euclid Avenue, Cleveland, OH 44106 USA; 3 Department of Mechanical and Aerospace Engineering, Case Western Reserve University, 10900 Euclid Avenue, Cleveland, OH 44106 USA; 4 Department of Orthopaedics, Case Western Reserve University, 10900 Euclid Avenue, Cleveland, OH 44106 USA

**Keywords:** Spinal cord injury, Gait, Hybrid neuroprosthesis, Exoskeleton, Functional neuromuscular stimulation, Biomechanics, Finite state machine, Assistive technology

## Abstract

**Background:**

Functional neuromuscular stimulation, lower limb orthosis, powered lower limb exoskeleton, and hybrid neuroprosthesis (HNP) technologies can restore stepping in individuals with paraplegia due to spinal cord injury (SCI). However, a self-contained muscle-driven controllable exoskeleton approach based on an implanted neural stimulator to restore walking has not been previously demonstrated, which could potentially result in system use outside the laboratory and viable for long term use or clinical testing. In this work, we designed and evaluated an untethered muscle-driven controllable exoskeleton to restore stepping in three individuals with paralysis from SCI.

**Methods:**

The self-contained HNP combined neural stimulation to activate the paralyzed muscles and generate joint torques for limb movements with a controllable lower limb exoskeleton to stabilize and support the user. An onboard controller processed exoskeleton sensor signals, determined appropriate exoskeletal constraints and stimulation commands for a finite state machine (FSM), and transmitted data over Bluetooth to an off-board computer for real-time monitoring and data recording. The FSM coordinated stimulation and exoskeletal constraints to enable functions, selected with a wireless finger switch user interface, for standing up, standing, stepping, or sitting down. In the stepping function, the FSM used a sensor-based gait event detector to determine transitions between gait phases of double stance, early swing, late swing, and weight acceptance.

**Results:**

The HNP restored stepping in three individuals with motor complete paralysis due to SCI. The controller appropriately coordinated stimulation and exoskeletal constraints using the sensor-based FSM for subjects with different stimulation systems. The average range of motion at hip and knee joints during walking were 8.5°–20.8° and 14.0°–43.6°, respectively. Walking speeds varied from 0.03 to 0.06 m/s, and cadences from 10 to 20 steps/min.

**Conclusions:**

A self-contained muscle-driven exoskeleton was a feasible intervention to restore stepping in individuals with paraplegia due to SCI. The untethered hybrid system was capable of adjusting to different individuals’ needs to appropriately coordinate exoskeletal constraints with muscle activation using a sensor-driven FSM for stepping. Further improvements for out-of-the-laboratory use should include implantation of plantar flexor muscles to improve walking speed and power assist as needed at the hips and knees to maintain walking as muscles fatigue.

## Background

Stepping has been restored in individuals with paralysis from spinal cord injury (SCI) using functional neuromuscular stimulation (FNS), passive lower limb bracing, powered lower limb exoskeletons, and different combinations of stimulation and bracing technologies. FNS can produce the majority of the joint torques needed to move or stabilize the lower extremities against collapse and enable users to stand up and walk [1–3]. In addition, stimulation provides added exercise benefits by contraction of the paralyzed muscles and allows the users to take advantage of their own muscle power to step [4]. On the other hand, passive lower limb bracing, such as reciprocal gait orthoses, are capable of providing controlled standing and coordinating the hip joints for stepping using upper body effort for individuals with SCI [5–8]. Powered lower limb exoskeletons use motors at the hip and knee joints to restore stepping with little or no active input from the users’ own muscles [9–22]. Thus, they require external power from batteries to perform all functional tasks such as standing up and stepping and use of upper extremities for balance.

Combining active stimulation of the users’ muscles with the supporting capabilities of exoskeletal bracing has highly desirable characteristics. In one system, a powered exoskeleton provided the appropriate amount of joint torque when extensor muscle activity generated by surface stimulation was unable to complete the desired stepping trajectory. The control system monitored muscle fatigue and optimized impedance control to allow the muscles to generate the movements instead of constraining the final knee movement to a defined trajectory. The system demonstrated the ability to coordinate surface stimulation with a motorized exoskeleton when evaluated in nondisabled volunteers and persons with incomplete SCI [23, 24].

Another system used an approach for cooperative control of surface stimulation of the hamstrings and quadriceps muscles with a lower extremity powered exoskeleton. The general control structure implemented a motor control loop to ensure tracking of the desired joint trajectories using joint angle feedback and a muscle control loop to adjust the surface stimulation such that motor torque contribution was minimized. The motor torque and power output of the exoskeleton with surface stimulation were reduced when compared to walking without stimulation in individuals with paraplegia [25].

The hybrid neuroprosthesis (HNP) is a muscle-driven exoskeleton which combines implanted FNS with passive controllable lower limb bracing. The neural stimulation and the resulting contractions of the otherwise paralyzed muscles generate the majority of required joint moments to move the limbs. The passive controllable bracing provides support, applies context-dependent constraints, and restricts degrees of freedom. The HNP enables individuals with SCI to stand, walk, negotiate stairs, and perform a controlled stand-to-sit transition maneuver [26–36]. By combining neural stimulation with an exoskeleton, upper limb effort was reduced by 42% and walking speed was increased by 15% as compared to conventional reciprocal gait orthoses. When compared to walking with neural stimulation alone, the HNP reduced both forward trunk tilt and upper limb loading by 17 and 36%, respectively [26]. The HNP also reduced stimulation duty cycle by more than two-thirds over walking with stimulation alone, potentially delaying the onset of fatigue and extending walking distance. This HNP system was tethered to the laboratory environment to run a closed-loop control system for walking and stair climbing and was not feasible for stepping in the community [26–33].

The implanted stimulation system of an HNP is also able to exercise the lower limb muscles when the exoskeleton is not used and provides added physiological benefits such as muscle and bone strength, bladder and bowel function, blood flow, and cardiovascular fitness that are not necessarily seen when standing with braces without stimulation [37–39]. When the individual user wants to walk longer distances, the implanted stimulation can be combined with the brace for stability and support.

The objective of this paper is to present results on the design and control of a new self-contained muscle-driven exoskeletal hybrid neuroprosthesis to restore walking in individuals with SCI in preparation for at-home and community use. When desired functional tasks (i.e. standing up or stepping) are to be performed, the power for movement is derived from stimulation of the paralyzed muscles not external energy from motors, and static joints are constrained by the exoskeletal bracing to maintain stability. The study sought to verify: 1) appropriate coordination of FNS with the lower limb bracing using the sensor-driven finite state controller, and 2) the ability to configure the hybrid neuroprosthesis to restore stepping in individuals with SCI using different combinations of surface and implanted stimulation systems.

## Methods

**Fig. 1:**
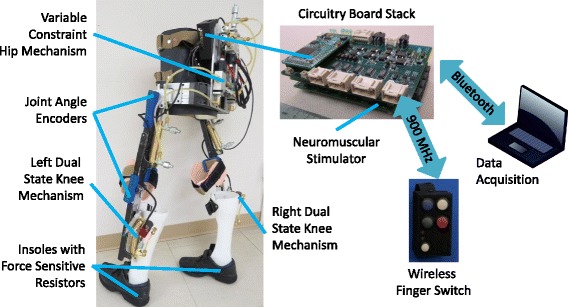
Self-contained exoskeleton architecture overview

The HNP consists of an instrumented exoskeleton with custom fitted corset and ankle foot orthoses (AFOs), adjustable uprights, and onboard hardware for controlling the exoskeletal joints and neural stimulation. In addition, the user interacted with the system via a wireless finger switch and a development interface designed for data collection and monitoring of the system in real time. The system components are shown in Fig. 1.

### Hardware

#### Exoskeleton

**Fig. 2:**
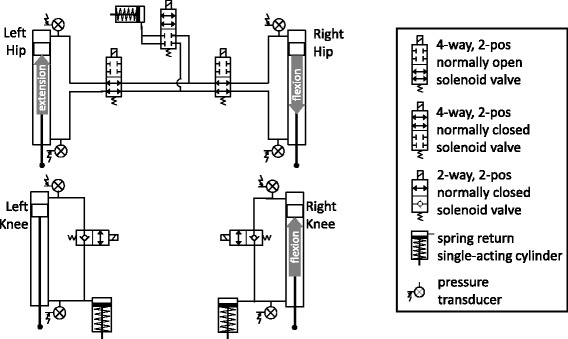
Hydraulic circuit schematics. *Top*: variable constraint hip mechanism (VCHM). *Bottom*: Dual state knee mechanisms (DSKMs). *Gray arrows* in the rod side of the cylinders indicate the direction of rod travel when the corresponding hip or knee joint flexion or extension occurs

The exoskeleton was designed with an adjustable pelvic band and uprights for a user of up to 100 kg and between 152 and 193 cm tall with attachments for a custom fitted corset and AFOs that keep the ankle joints fixed at neutral. Attached to the exoskeleton (7.3 kg) were hydraulic mechanisms (7.9 kg) at the knees and hips. A variable constraint hip mechanism (VCHM) provided hip-hip reciprocal coupling as well as independent locking and unlocking of the hip joints (Fig. 2) [26, 40]. The hip-hip reciprocal coupling provided postural trunk support and hip flexion by coupling with contralateral hip extension. Dual state knee mechanisms (DSKMs) independently locked and unlocked the knee joints [27, 28]. The hydraulic mechanisms were designed to make the exoskeleton function as a reciprocal gait orthosis when unpowered or in case of power failure to ensure safety. Bench testing was performed to determine the average passive resistance of the hydraulic circuits for angular velocities up to 120 deg/s, where passive resistance is the torque needed at a joint to move the system at a specific angular velocity. The passive resistance of the hip joint was 7.8 Nm in flexion and 10.5 Nm in extension during hip independent motion, and 8.4 Nm in flexion and 11.9 Nm in extension during hip-hip reciprocal coupling motion. The average passive resistance of the DSKM was 2.6 Nm in flexion and 2.4 Nm in extension. The unlocking response time of the DSKMs was 43–200 ms, depending on the amount of load on the knee [28].

#### Sensors

The exoskeleton was instrumented with sensors for closed-loop control that include: joint encoders (US Digital, Vancouver, WA, USA) and force sensitive resistors (B & L Engineering, Tustin, CA, USA). Encoders were installed bilaterally at the hip and knee joints on the exoskeleton to measure joint angles. The insoles with force sensing resistors (FSRs) were placed in the shoes to monitor floor contact pressure under the 1^st^ and 5^th^ metatarsals, 1^st^ phalange, and heel. An emergency switch was included for safety in the event that stimulation needed to be disabled. The user interacted with the system via a custom wireless finger switch to indicate the desire to stand up, sit down, or take a step. Commands from a combination of the sensor signals triggered transitions between states in the finite state machine (FSM) controller to progress through the gait cycle.

#### Controller hardware

**Fig. 3:**
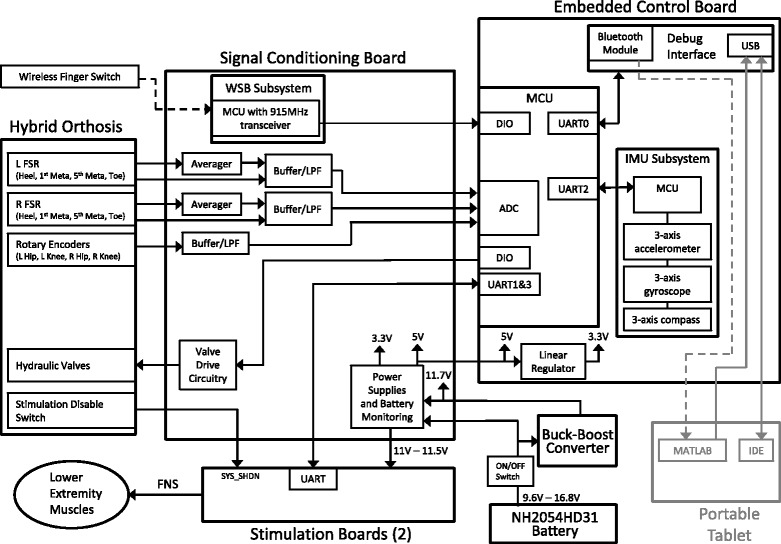
HNP system block diagram. (Meta: metatarsal joint of foot; L: left; R: right; SYS_SHDN: system stimulation shutdown signal; LPF: low pass filter; MCU: microcontroller unit; WSB: wireless sensor board)

The onboard control system hardware included a signal conditioning board (SCB), an embedded control board (ECB), two stimulation boards, and a power supply (0.7 kg) (Fig. 3). The SCB averaged together the three forefoot FSR signals from each insole. The SCB then low pass filtered (at 25 Hz) the encoder, heel FSR, and averaged forefoot FSR sensor signals. The SCB also generated the drive signals for the hydraulic valves. The ECB contained an ATmega2560 microcontroller (Atmel Co., San Jose, CA, USA), which sampled the analog sensor signals up to 50 Hz and generated the digital valve and stimulation control signals. The ECB also contained the Bluetooth module and an inertial measurement unit (IMU) subsystem. The IMU subsystem could be used as an alternative signal or in combination with the other sensor signals for step initiation or balance control, similar to commercial exoskeleton systems [14]. The ECB communicated with a laptop/personal computer either wirelessly via Bluetooth or through a USB port for software development, controller tuning, and data acquisition for post-processing. There were three combinations of two stimulation boards used for testing in three subjects: 1) two 12-channel percutaneous stimulation boards that sent stimulus pulses to through-the-skin intramuscular electrodes, 2) one implant controller board for a 16-channel implanted stimulator telemeter (IST) and one 4-channel bipolar surface stimulation board, and 3) one implant controller board for a 16-channel IST implant and one implant controller board for an 8-channel implanted receiver-stimulator (IRS) implanted pulse generator (IPG). Other combinations of boards for stimulation can be configured depending on the user’s needs. Each percutaneous stimulation board was capable of providing up to 12 channels of stimulation at amplitudes up to 20 mA and pulse widths up to 250 μs. Each IST pulse generator was capable of providing up to 16 channels of stimulation at amplitudes up to 20 mA and pulse widths up to 250 μs, depending on whether an intramuscular or cuff electrode was used for a particular channel [41, 42]. The IRS pulse generator was capable of providing up to 8 channels of stimulation at amplitudes up to 20 mA and pulse widths up to 250 μs [43]. The surface stimulation board was able to provide up to 4 bipolar channels of stimulation with amplitudes up to 100 mA and pulse widths up to 250 μs. Power for the HNP system was provided by an Inspired Energy NH2054HD31 lithium ion battery pack (14.4 V, 6.2 Ah, 0.44 kg, 15.24 cm × 7.9 cm × 2.3 cm). A regulated 11.7 V supply was generated by a buck-boost converter to power the HNP valves and be used by the SCB to generate the other voltages necessary for the electronics (e.g., 3.3 V, 5 V). Based on electrical current draw for the system during stepping, the battery power was estimated at 2 h between charging. The SCB, ECB, stimulation boards, and power supply were all mounted on the exoskeleton.

### Software control system

The closed-loop FSM controller used the information from the sensors to output the appropriate neural stimulation and set hydraulic joint constraints. High-level control of the FSM defined the functions of the hydraulic system and pre-programmed stimulation patterns for the *sit-to-stand*, *standing*, *stepping*, and *stand-to-sit* functions heuristically tuned for each participant based on his muscle set and implanted system configuration. Within the *stepping* function, the FSM switched between phases of gait and delivered the appropriate left or right step stimulation.

**Fig. 4:**
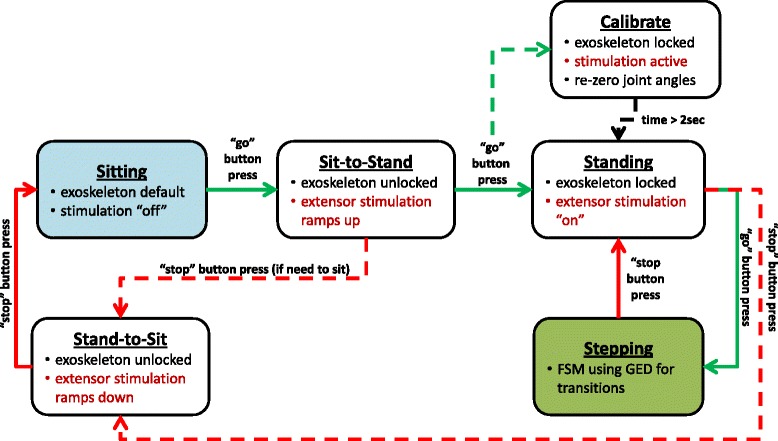
High-level control system that initially began in the sitting function and transitioned user selected functions. Stimulation is indicated in *red font color*, *black arrows* indicate transitions that occur based on thresholds, *green arrows* correlate to pressing the “go” button, and *red arrows* correlate to pressing the “stop” button

The high-level control transitioned between the different functions of *sitting*, *sit-to-stand*, *standing*, *stepping*, and *stand-to-sit* (Fig. 4). The transitions were initiated by pressing different buttons on the wireless finger switch. The user was initially in the *sitting* function with the hips reciprocally coupled to provide truncal stability, knees locked at 110°, and all stimulation turned off. The *sit-to-stand* function was initiated by pressing the “go” button which uncoupled the hips, unlocked the knee joints, and maximally activated hip and knee extensor muscles to power the user into standing. Once the user was in an upright posture with all joints at neutral (*standing* function), all joints were locked with a “go” button press and the joint angle encoders were calibrated by pressing the “calibrate” button. From the *standing* function, the user had a choice to transition to stepping or to return to sitting. The *stand-to-sit* function was activated by pressing the “stop” button that unlocked all joints and ramped down the stimulation to the hip and knee extensor muscles.

**Fig. 5:**
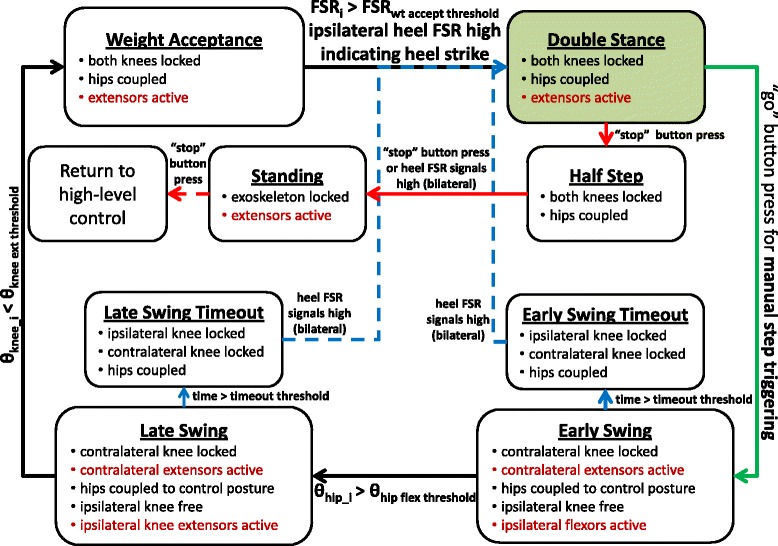
FSM using GED within the stepping function to transition through phases of gait. Transition between early swing and late swing occurred when the ipsilateral hip joint angle (θ_hip_i_) exceeded a predetermined hip flexion angle threshold (θ_hip flex threshold_). Transition between late swing and weight acceptance occurred when the ipsilateral knee joint angle (θ_knee_i_) reached extension as indicated by being less than the set knee extension joint angle threshold (θ_knee ext threshold_). Before the user could initiate the next step, the ipsilateral heel FSR signal (FSR_i_) had to exceed the set weight acceptance threshold (FSR_wt accept threhold_). Stimulation is indicated in *red font color*, *black arrows* indicate transitions that occur based on thresholds in GED, *blue arrows* indicate transitions in Timeout phases, *green arrow* correlates to pressing the “go” button, and *red arrows* correlate to pressing the “stop” button. (i = ipsilateral)

After entering the *stepping* function, the controller alternated between left and right steps. A gait event detector (GED) identified appropriate transitions between phases in the stepping function of the FSM based on sensor signals. In particular, the GED determined transitions between *double stance (before left swing), left early swing, left late swing, left weight acceptance, double stance (before right swing), right early swing, right late swing, and right weight acceptance* phases of gait (Fig. 5). Sensor thresholds for the GED were initially selected from testing in nondisabled volunteers and heuristically adjusted as necessary for the subject with SCI. *Double stance*, *early swing*, *late swing*, and *weight acceptance* phases alternated between left and right legs. During *left early swing* phase, the right (contralateral) knee was locked and the left (ipsilateral) knee was unlocked, and vice versa during *right early swing* phase. *Double stance* phase was characterized by both ipsilateral and contralateral heels/forefoot being in contact with the ground. During *double stance*, all hydraulic valves were in their default state for hip-hip reciprocal coupling and locked knees. Once the subject initiated the next step by pressing the “go” button, the FSM transitioned to *early swing* phase where stimulation for the swing leg was initiated. The knee mechanism unlocked to allow the knee to swing freely while the hips remained reciprocally coupled and the contralateral stance knee remained locked. When the swing leg hip flexion angle exceeded a predetermined threshold in *early swing* phase, the FSM transitioned to *late swing* phase with knee extensor stimulation. Once the knee exceeded an extension threshold, the knee was locked in preparation for *weight acceptance* phase. When heel contact of the advanced leg exceeded the FSR heel threshold, the FSM transitioned from *weight acceptance* to *double stance. Weight acceptance* phase ensured that weight was transferred to the stance leg before the subject initiated another step. The subject then initiated the next step for the opposite leg by pressing the “go” button [36].

*Timeout* phases were incorporated into the FSM for safety. The FSM transitioned to the *timeout* phases if the hip or knee angle thresholds during swing were not achieved within a prescribed time determined by the lengths of the pre-programmed stepping stimulation pattern. In the *timeout* phase, the exoskeleton returned to default reciprocally coupled hips and locked knees while maximally activating the hip and knee extensor muscles. To exit the *stepping* function of the FSM, the subject pressed the “stop” button to return to the *standing* function before initiating the *stand-to-sit* maneuver with all joints freed and stimulation ramping down.

### Subject testing

Three male individuals with motor complete SCI volunteered and signed the written consent forms to participate in this study approved by the Louis Stokes Cleveland Department of Veterans Affairs Medical Center Institutional Review Board to walk with the HNP system. The participants ranged in age from 54 to 59 years, with a mean height of 181 ± 10 cm and mean weight of 78 ± 14 kg. Subject A used two percutaneous stimulation boards, Subject B used one IST-16 and one surface stimulation board, and Subject C used one IST-16 and one IRS-8 board. Each subject had a slightly different muscle set, but all subjects had electrodes implanted to contract muscles for hip extension (posterior portion of adductor magnus, gluteus maximus, or hamstrings) and knee extension (vastus intermedius, vastus medialis, vastus lateralis, or a combination of quadriceps). Subject A also had percutaneous intramuscular electrodes implanted bilaterally to activate hip flexor muscles (tensor fasciae latae, sartorius), knee flexor muscles (gracilis, sartorius), and ankle dorsiflexor muscle (tibialis anterior). Subject B used surface electrodes to activate the common peroneal nerve for the withdrawal reflex for hip and knee flexion and ankle dorsiflexion. Subject C had electrodes bilaterally implanted to activate the hip flexor muscle (iliopsoas) and ankle dorsiflexor muscle (tibialis anterior), with additional hip flexor muscles (tensor fasciae latae and sartorius) and knee flexor muscle (sartorius) on the right side. The posterior portion of the adductor magnus and gluteus medius were implanted to provide medial-lateral stability when using stimulation alone. The subject characteristics and muscles stimulated are listed in Table 1.

**Table 1:** Characteristics of participants

	Subject A	Subject B	Subject C
Sex	M	M	M
Age (years)	54	56	59
Weight (kg)	64	92	79
Height (cm)	175	193	175
Injury Level	T4	T11	T4
AIS	AIS A	AIS B	AIS B
Time Since Injury (years)	32	7	8
Time Since Implant (years)	31	4	4
Stimulation Boards Used	Two percutaneous	One IST-16 and one bipolar surface	One IST-16 and one IRS-8
Type of electrode (# of electrodes)	Percutaneous intramuscular (16)	Intramuscular (8) Nerve cuff (2) Surface (2)	Intramuscular (18) Nerve cuff (6)
Muscles Stimulated	PA, GM, VI, VM, VL, QD, TFL, ST, GR, TA	PA, GM, ME, HS, QD, WD	PA, GM, ME, HS, QD, TFL^a^, ST^a^, IL, TA

*AIS* American Spinal Injury Association Impairment Scale, *C* cervical, *GM* gluteus maximus, *GR* gracilis, *HS* hamstrings, *IL* iliopsoas, *ME* gluteus medius, *PA* posterior portion of adductor magnus, *QD* quadriceps, *ST* sartorius, *T* thoracic, *TA* tibialis anterior, *TFL* tensor fasciae latae, *VI* vastus intermedius, *VM* vastus medialis, *VL* vastus lateralis, *WD* withdrawal reflex

^a^Right side only

The exoskeleton uprights were adjusted to fit the leg lengths of the subject such that the hip and knee joint centers were aligned. The participant transferred to a chair that had the exoskeleton uprights abducted for ease of donning. The subject was then strapped into the thoracic corset, the AFOs and to the uprights.

**Fig. 6:**
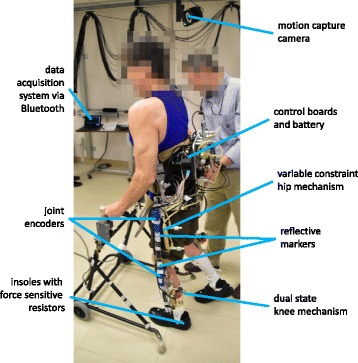
Experimental setup for subject testing

Testing was performed along the 10 m walkway within the view of a 16-camera Vicon MX40 digital motion capture system (Vicon, Inc., Oxford, UK) to measure walking speed and cadence (Fig. 6). Reflective markers were attached bilaterally on the subject’s shoes (calcaneous, 2^nd^ metatarsal head) to determine gait speed and cadence. Rotary encoders measured joint angles and FSRs recorded floor contact. Subjects used a two-wheeled walker for balance and a standby assist for safety.

The subjects initiated *sit-to-stand* by pressing the “go” button on the wireless finger switch to activate the hip and knee extensor muscles via stimulation. Once upright, the hip and knee joints were locked and the joint angles calibrated. Stimulation of the extensor muscles remained “on” when in the *standing* function to accommodate for any destabilizing play in body to exoskeleton coupling. The subject pressed the “go” button to initiate *stepping* which coordinated the stimulation with exoskeleton constraints based on the FSM. Each subject walked at his preferred speed, which was influenced by timing of the neural stimulation. During walking, the hips remained reciprocally coupled to maintain posture while the knees locked or unlocked depending on the phase in the FSM. Hip and knee angles, FSR signals, and function or gait phase transitions were transmitted wirelessly over Bluetooth and recorded on a portable tablet during the walking trials for post-processing. Onboard data were sampled at 50 Hz, and laboratory motion capture data were collected at 200 Hz. Outcome measures included average change in hip and knee angles, walking speed, and cadence.

## Results

**Fig. 7:**
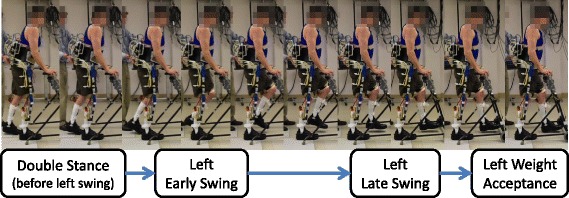
Left step progression for Subject A during gait

**Fig. 8:**
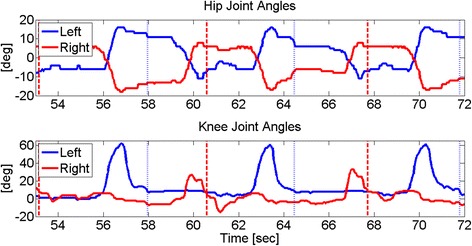
Hip and knee flexion (+) and extension (-) for Subject A. *Vertical lines* indicate left (*blue dotted*) and right (*red dashed*) heel strike

All participants were able to walk with the self-contained HNP system without prior practice. The subjects pushed the walker forward before initiating a step. After pressing the “go” button, the knee mechanism was unlocked, stimulation to the quadriceps muscles was disabled, and the hip and knee flexor muscles were activated via stimulation while the hip mechanism remained coupled. Once the knee was extended with stimulation during swing, the knee joint was locked to prepare for weight acceptance. An example of left step and joint angle data for Subject A is illustrated in Figs. 7 and 8. A total of at least 22 left and right steps were collected and analyzed for each subject, with the results summarized in Table 2. Subject A achieved sufficient foot-floor clearance during walking, as indicated by an average (± standard deviation) change in knee angle of 43.6° ± 11.1°. Subjects B and C had some difficulty achieving sufficient knee flexion for good floor clearance, having average changes in knee angle of 14.0° ± 4.9° and 19.2° ± 11.0°, respectively. The limited average change in hip angle as reported in Table 2 was expected as the hips were continuously reciprocally coupled during stepping. Walking speeds ranged between 0.03 and 0.06 m/s and step cadences were between 10 and 20 steps/min.

**Table 2:** Gait outcome measures for each subject

Subject (*n* = # of steps)	A (*n* = 28)	B (*n* = 22)	C (*n* = 75)
Average change in hip angle (± std) [degrees]	19.1 ± 4.5	8.5 ± 2.0	20.8 ± 7.4
Average change in knee angle (± std) [degrees]	43.6 ± 11.1	14.0 ± 4.9	19.2 ± 11.0
Speed [m/s]	0.06	0.03	0.05
Cadence [steps/min]	20	10	14

## Discussion

Three subjects with motor complete SCI were evaluated with the untethered stimulation-driven exoskeleton. The original goals of coordinating FNS with controllable lower limb bracing and customizing to users’ implanted stimulation systems were found to be feasible. The onboard control system was successfully configured for different stimulation systems for each subject and appropriately coordinated FNS to contract the lower limb muscles with the passive exoskeletal brace constraints.

Subject A, who had a percutaneous implanted stimulation system for walking, achieved sufficient hip and knee flexion to consistently clear his foot during swing. On the other hand, Subjects B and C had stimulation systems implanted for standing and some minimal stepping in the vicinity of the wheelchair. Subject B, who used surface stimulation of the common peroneal nerve to elicit the flexion withdrawal reflex, had only marginal hip and knee flexion, often resulting in foot drag during swing. The flexion withdrawal reflex via the common peroneal nerve appeared to habituate over time and resulted in a decreased reflex response [44]. Even though Subject C had implanted hip flexors bilaterally and hip and knee flexors on the right, he used upper extremities for hip hiking to achieve sufficient clearance for the swing leg. This subject experienced knee extensor stiffness which affected his stepping in the exoskeleton. In general for all subjects, there were no differences in the joint angle changes between the early steps taken and the later steps that would indicate muscle fatigue from FNS. However, since some major muscles for walking were not available for stimulation or produced marginal strength to maintain motion and overcome the weight and resistance of the exoskeleton, future systems will require power assist as needed for long distance walking.

While we had no direct measurement of joint torques during walking, stimulated moments generated at the hip and knee were sufficient for short distance walking with the exoskeleton. In our previous study where the hydraulic system was instrumented with pressure sensors, we were able to calculate joint torques. The peak torques at the hip and knee were 12 and 20 Nm, respectively [40]. Thus, the torques generated by the muscles can be estimated to be the measured torque plus the passive resistive torque of the hydraulics. We know that the passive resistance and the weight of orthosis had an effect on the walking speed since Subject A was able to walk much faster with stimulation alone. In addition, nondisabled individuals walking with the same configuration of the exoskeleton consumed about twice as much O_2_/kg/min as when walking at the same speed without the exoskeleton. In addition, in previous studies we reported on the hip flexion and extension torques of approximately 60 Nm generated by the implanted stimulation system where the steady state torques were reached in 3 min and settled at approximately 30% of initial value [1]. These values suggest the need for external power assist as needed for practical walking distances and durations.

The response time to unlock the knee mechanism (43–200 ms) and delay from the onset of stimulation to the generation of the desired muscle force (100 ms) could potentially influence the coordination of the FNS and exoskeleton constraints during walking, resulting in slower gait. The stimulation of the flexor muscles to complete a step for all subjects was delayed by a minimum of 200 ms after the “go” button was pressed, allowing time for the knee mechanism to unlock before muscle force was generated via stimulation. Designing mechanisms with minimal unlock response time could be beneficial for reducing the delay after step initiation to increase walking speed in a muscle-driven HNP. In addition, passive resistance of the hydraulic mechanisms can slow the system and should be reduced.

There is a potential for physiological gains in individuals with SCI by combining the muscle-driven aspects of this design with the currently commercialized exoskeletons. We demonstrated feasibility that a participant’s internal torque generating capabilities from stimulated muscle can enable stepping in combination with a passive controllable lower limb exoskeleton. In other approaches, powered exoskeletons were combined with neural stimulation to reduce external power consumption and to provide physiological benefits [23–25]. To optimize the physiological benefits, muscle contraction can be used as the primary source of power for ambulation and external motors, implemented with minimal additional passive resistance, could provide assist as-needed when muscles are weakened or fatigued.

The participants walked with an average speed of 0.05 m/s and cadence of 15 steps/min. Variability in the speeds and cadences between the subjects may be due to differences in muscle strength, muscles stimulated, and lack of practice walking with the hybrid system. These speeds and cadences are slower compared to those measured with individuals walking with stimulation alone (0.5–0.9 m/s) [1–3], reciprocal gait orthoses (0.1–0.3 m/s) [5–8], or motorized exoskeletons (0.1–0.5 m/s) [12–14, 20, 45, 46].

The walking speed and cadence for the participants using the muscle-driven exoskeleton system were relatively low. With external motor power, exoskeleton average walking speed was 0.26 m/s and maximum walking distances during standard 6-min walking tests were between 121 and 171 m [12–14, 20, 45, 46]. Individuals with SCI walking with stimulation alone walked at speeds ranging from 0.5 to 0.9 m/s over maximum distances of 300–400 m before sitting down due to muscle or cardiovascular fatigue [1–3]. To achieve the same or similar walking speeds, assistance could be provided at the ankle with the addition of plantar flexor stimulation [1, 2, 47] or with bursts of power from small motors [22]. The ankle moment generated by plantar flexion provides an active weight transfer with increased step length and speed of walking [48]. Assistive power on an as-needed basis could enhance the joint moments needed to complete the step when the stimulated muscles begin to fatigue [23, 24]. The next generation of the muscle-driven exoskeleton under development includes small motors to provide power assist as needed to enable individuals who may not have adequate stimulated output from the paralyzed muscles. Combining plantar flexor stimulation with the powered exoskeletons, or combining plantar flexor stimulation and small motors with the muscle-driven exoskeleton may help users achieve walking speeds in excess of 0.5 m/s and distances of 600 m for community ambulation in the future [49].

## Conclusion

A self-contained muscle-driven exoskeleton is feasible to restore stepping in individuals with motor complete paralysis from SCI. A finite state controller using a sensor-based gait event detector was able to coordinate the power for movement provided by the neural stimulation with controllable constraints of the exoskeleton to provide stability. Future work will investigate the addition of plantar flexor stimulation and small motors to enhance joint moments on an assist as-needed strategy to increase gait speed, cadence, and walking distance.

## Abbreviations

AFOs: Ankle-foot orthoses
DSKM: Dual state knee mechanism
ECB: Embedded control board
FNS: Functional neuromuscular stimulation
FSM: Finite state machine
FSR: Force sensitive resistor
GED: Gait event detector
HNP: Hybrid neuroprosthesis
IMU: Inertial measurement unit
IPG: Implanted pulse generator
IRS: Implanted receiver-stimulator
IST: Implanted stimulator telemeter
LPF: Low pass filter
SCB: Signal conditioning board
SCI: Spinal cord injury
VCHM: Variable constraint hip mechanism
